# A133 QUALITY INDICATOR ADHERENCE FOR BARRETT’S ESOPHAGUS: A RETROSPECTIVE ANALYSIS

**DOI:** 10.1093/jcag/gwae059.133

**Published:** 2025-02-10

**Authors:** C Roda, D Koerber, A Walia, P Tavakoli, E Taylor, M Rui Xuan Yu, S Pang, D Motomura, E Lam, R Enns, W Xiong, N Shahidi

**Affiliations:** Gastroenterology, The University of British Columbia Faculty of Medicine, Vancouver, BC, Canada; Gastroenterology, The University of British Columbia Faculty of Medicine, Vancouver, BC, Canada; St Paul’s Hospital, Vancouver, BC, Canada; St Paul’s Hospital, Vancouver, BC, Canada; St Paul’s Hospital, Vancouver, BC, Canada; Gastroenterology, The University of British Columbia Faculty of Medicine, Vancouver, BC, Canada; St Paul’s Hospital, Vancouver, BC, Canada; St Paul’s Hospital, Vancouver, BC, Canada; St Paul’s Hospital, Vancouver, BC, Canada; St Paul’s Hospital, Vancouver, BC, Canada; St Paul’s Hospital, Vancouver, BC, Canada; St Paul’s Hospital, Vancouver, BC, Canada

## Abstract

**Background:**

Consensus guidelines advocate for quality indicator adherence in patients with Barrett’s Esophagus (BE). This includes documentation of landmarks/extent of BE, adherence to biopsy protocols and recommended surveillance intervals.

**Aims:**

Evaluate adherence to established quality indicators for patients with BE in a tertiary referral centre.

**Methods:**

Between 01/2012 - 01/2024, consecutive patients with intestinal metaplasia with goblet cells on esophageal biopsies were identified using a validated histopathology registry at St. Paul’s Hospital (Vancouver, BC, Canada). After confirming the diagnosis of BE (≥ 1cm of columnar epithelium above the top of the gastric folds on index esophagogastroduodenoscopy), quality indicator adherence was evaluated (documentation of landmarks/extent of BE (Prague Classification), adherence to biopsy protocols (Seattle Protocol) and recommended surveillance intervals). Continuous variables were summarised using median (IQR). Categorical variables were summarised as frequencies (%).

**Results:**

Preliminary analysis between 01/2012 - 03/2015 identified 253 patients with BE. Median age was 61 years (IQR 17), with 196 (77.5%) being male. Of these, 81 patients were diagnosed with neoplastic BE (33.3% referred for neoplastic BE management; 24.7% at index esophagogastroduodenoscopy; 42.0% during surveillance). The median time to low-grade dysplasia, high-grade dysplasia and esophageal adenocarcinoma from BE diagnosis was 12 months (IQR 48 months), 13 months (IQR 47 months) and 48 months (IQR 33 months), respectively. At index or follow-up evaluation, landmark delineation/extent of BE was documented in 23.3%. Biopsy protocol adherence was 86.6%. Adherence to surveillance recommendations was 57.7%.

**Conclusions:**

Quality Indicators for BE are poorly adhered to, outside of established biopsy protocols. Programmatic BE management may carry the potential to improve patient outcomes and resource utilization.

**Funding Agencies:**

None

